# Application value of the vertical and horizontal hierarchical responsibility management nursing model in patients undergoing appendectomy

**DOI:** 10.3389/fsurg.2025.1652237

**Published:** 2025-09-29

**Authors:** Jingjing Miao, Qianqian Zhang, Huimin Li

**Affiliations:** The Second Department of General Surgery, Funan County People’s Hospital, Fuyang, Anhui, China

**Keywords:** appendicitis, appendectomy, vertical and horizontal hierarchical responsibility, postoperative recovery, nursing satisfaction

## Abstract

**Background:**

Appendicitis is a common acute abdominal disease with characteristic symptoms. The vertical and horizontal hierarchical responsibility management nursing model strengthens nursing staff training, refines nursing work contents, and contributes to improved patient care during hospitalization.

**Objective:**

This study aimed to evaluate the application value of the vertical and horizontal hierarchical responsibility management nursing model in patients undergoing appendectomy.

**Methods:**

A total of 86 acute appendicitis patients undergoing appendectomy in our hospital from January 2021 to January 2023 were selected as research subjects. All patients were assigned to a responsibility group and a control group using the random number table method, with 43 patients in each group. The control group underwent routine nursing intervention, whereas the responsibility group underwent management and nursing with a vertical and horizontal hierarchical responsibility system.

**Results:**

First postoperative mealtime, first postoperative exhaust time, postoperative hospitalization time, and first postoperative out-of-bed activity time in the responsibility group depicted depletion relative to those of the control group (*p* < 0.05). The total nursing satisfaction rate in the responsibility group depicted an elevation relative to that in the control group post-nursing care (*p* < 0.05). The incidence of complications in the responsibility group 14 days post-surgery depicted depletion relative to that in the control group (*p* < 0.05). The QOL scores in both groups post-nursing care depicted elevation relative to before, and the responsibility group depicted elevation relative to that in the control group (*p* < 0.05).

**Conclusion:**

The application of the vertical and horizontal hierarchical responsibility management nursing model in appendectomy patients can improve clinical nursing efficacy and facilitate patient recovery, which is worthy of clinical application.

## 1 Introduction

Appendicitis is a common acute abdominal disorder, characterized by rapid disease progression, and delayed treatment often leads to systemic inflammatory response syndrome and shock (1). Once diagnosed, timely appendectomy is the key treatment for appendicitis (2). In recent years, with the rapid development of minimally invasive techniques, the success rate of appendectomy has been increasing significantly (3). Moreover, appendectomy has several advantages including ease of operation, shorter surgical duration, fast postoperative recovery, safety and reliability, and reduced postoperative pain for patients (4, 5). However, surgical success is only the first step toward patient recovery. Postoperative care during appendectomy plays a crucial role in the patient's overall recovery (6). Many patients experience complications such as malnutrition and immune dysfunction after the surgery, which can hinder postoperative recovery and increase the risk of postoperative complications (7). Postoperative malnutrition may result from temporary disruptions in digestive and absorptive functions caused by surgical stress, as well as reduced food intake due to pain from the wound (8). Meanwhile, immune dysfunction is related to the body's stress response and the release of inflammatory mediators triggered by surgical trauma. When immune function declines, energy consumption increases, and the risk of infection rises, creating a vicious cycle within the body (9). In such cases, effective postoperative care plays a crucial role. Through scientific and reasonable dietary care, providing patients with sufficient and balanced nutritional support helps improve malnutrition conditions and enhance their body's resistance; meticulous wound care can prevent infections and reduce the risk of complications; and comprehensive rehabilitation guidance, including encouraging patients to engage in appropriate early activities, can promote the recovery of physical functions and break the vicious cycle of declining immune function, increased energy consumption, and rising infection risks (10). Therefore, in-depth exploration of the relevant content of postoperative care after appendectomy is extremely significant to ensure the smooth recovery of patients and improve overall surgical efficacy.

In recent years, people have become increasingly concerned about health knowledge and have gradually raised their requirements for the quality of medical care (11). New concepts, new ideas, and new technologies in the field of nursing are also constantly emerging (12). Currently, in clinical practice, the mastery of nursing knowledge is generally still at a basic level, and the understanding of humanistic nursing knowledge is also relatively lacking (13). At the same time, routine nursing intervention measures have certain deficiencies in preventing complications and providing psychological care, lacking the necessary content and characteristics of nursing (14). The vertical and horizontal hierarchical responsibility management nursing model refers to comprehensive nursing provided by a specialized nursing staff from the moment of patients' admission until discharge, providing personalized, holistic, and continuous nursing intervention for patients (15). It has been reported that Connect, Introduce, Communicate, Ask, Respond, Exit (CICARE) and hierarchical responsibility management nursing model coordination can effectively improve the cardiac function levels and significantly enhance their exercise tolerance, self-efficacy, and quality of life in elderly patients with chronic heart failure (16). However, the application effect of a vertical and horizontal hierarchical responsibility management nursing model in patients undergoing appendectomy remains unclear.

We specifically evaluated the application value of the vertical and horizontal hierarchical responsibility management nursing model in patients undergoing appendectomy. The current report is as follows.

## 2 Methods

### 2.1 Universe and sample

This was an intervention study. Based on the inclusion and exclusion criteria, a total of 86 acute appendicitis patients who underwent appendectomy at Funan County People's Hospital from January 2021 to January 2023 were selected as research participants. All patients were assigned to a responsibility group and a control group using the random number table method, with 43 patients in each group. This research was approved by the Medical Ethics Committee of our hospital. All research subjects were informed of the research contents and signed informed consent.

### 2.2 Inclusion and exclusion criteria

Inclusion criteria: patients aged 18–75 years old; stable condition consistent with the diagnosis of acute appendicitis (17); time from onset to surgery less than 12 h; no surgical contraindications such as severe cardiopulmonary diseases or multiple organ dysfunctions before surgery; complete clinical data. Exclusion criteria: patients with respiratory infectious diseases; those who had taken or injected immunosuppressive drugs within 1 month before admission; those with congenital immune system diseases; those with tumors; or women who are pregnant, planning to conceive, or breastfeeding.

### 2.3 Sample size calculation

In this study, we conducted a power analysis using the G*Power 3.1.9.7 software to determine the sample size required to detect the statistical differences. With an *α* value of 0.05 and a 90% power analysis, the analysis indicated that 43 patients were needed in each group. Therefore, to draw reliable conclusions, the study sample size was set at 43 patients per group.

### 2.4 Implementation of the research

The control group underwent routine nursing intervention. The nursing staff were instructed to closely monitor changes in postoperative patients' vital signs, and their knowledge of routine nursing practices for acute appendicitis should be regularly assessed. Any nursing staff who did not have a proper understanding should be punished accordingly.

The responsibility group underwent management and nursing with a vertical and horizontal hierarchical responsibility system, and specific measures are as follows. (1) Strengthen training of nursing staff. The first step was for nursing staff to learn about the vertical and horizontal hierarchical responsibility system. The learning content mainly focused on specific requirements, significance, and objectives of the vertical and horizontal hierarchical responsibility system, further strengthening the service concept of humanistic service and full process nursing. The training was provided for nursing staff, who were required to communicate with each other, and targeted training was provided on any issues that may arise during the communication process. The training was conducted in the hospital's academic lecture hall. This training was open to all the nursing staff of this responsibility group, with a total of 20 nursing staff participating. Taking into account the systematic nature of the training content and the learning characteristics of the nursing staff, we adopted a combination of group training and centralized training. During the centralized training phase, experienced experts provided comprehensive and systematic explanations, enabling the nursing staff to have an overall understanding and comprehension of the vertical and horizontal hierarchical responsibility system. Subsequently, based on factors such as the position and work experience of the nursing staff, they were reasonably grouped, with approximately 10 nursing staff in each group. Group training facilitated deeper communication and discussion among the nursing staff and involved case analysis and simulation exercises to address potential problems encountered in actual work, thereby enhancing the training's relevance and effectiveness. To ensure the effectiveness of the training, we adopted a combined approach of multiple training methods, including oral explanations, written materials, video demonstrations, and simulation exercises. The entire training period lasted for 4 weeks, with one training session held each week, lasting approximately 2 h. (2) Refine work content. According to the requirements of routine nursing, contents of graded nursing need to be refined, work processes be optimized, work systems and job responsibilities be improved, and quality standards be established. (3) Scientifically implement vertical and horizontal hierarchical management. Different nursing interventions should be assigned to responsible nursing staff and general responsible nursing staff based on different educational backgrounds and abilities of patients, and integrated nursing and grouping management should be provided for all patients. The work with relatively high technical content and complexity is subject to nursing intervention by general responsible nursing staff, while responsible nursing staff is required to provide full nursing intervention and supervise the effectiveness and efficacy of nursing work. (4) Adjust scheduling method. The scheduling should consider patients' actual situation, their condition, and the thoughts of the nursing staff for reasonable scheduling. Simultaneously, the scheduling sequence should be adjusted appropriately at any time, and nursing should be carried out with a planned and targeted approach; two people are required to be on duty for the night shift and noon shift. Each patient should be managed by a dedicated nursing staff, and each patient also should be taken care of during break time; when changing shifts, patients' specific situations should be clearly explained to on on-duty nursing staff to prevent nursing work interruptions that may cause poor prognosis for surgical patients. (5) Interdepartmental cooperation and support should be strengthened as much as possible. Support between different departments should be further strengthened, performance allocation systems should be improved, bonus support should be appropriately elevated, and the nursing staff's proactive cooperation and service awareness should be enhanced. Besides, the workflow of each department should be improved, the nursing staff workflow and nursing documents should be simplified, and the time for nursing staff to serve patients should be improved. (6) The supervision and inspection mechanism needs to be further improved. Relevant supervision and management equipment should be improved, and the contents of various basic nursing service projects should be classified, prioritized, and publicized. Simultaneously, it is necessary to provide corresponding supervision and guidance for nursing work and implement the expected outcomes of the task.

### 2.5 Data collection tools

(1) First postoperative mealtime, first postoperative exhaust time, postoperative hospitalization time, and first postoperative out-of-bed activity time in both groups were recorded. (2) A self-designed questionnaire developed by our hospital was applied to assess the nursing satisfaction post-nursing care. The ratings included significant satisfaction, satisfaction, and dissatisfaction. A score of >80 indicated significant satisfaction, 60–80 indicated satisfaction, and <60 indicated significant dissatisfaction. Total nursing satisfaction rate = (significant satisfaction + satisfaction) cases/total cases × 100%. (3) Telephone follow-up was conducted on discharged patients to record the occurrence of complications such as intestinal obstruction, incision infection, and abdominal abscess in both groups 14 days post-surgery. Total complication rate = (intestinal obstruction + incision infection + abdominal abscess) cases/total cases × 100%. (4) Post-nursing care, patients' quality of life (QOL) was evaluated through the MOS 36-item Short Form Health Survey (SF-36). This questionnaire is commonly used in clinical trials to assess general physical and mental health and has demonstrated construct validity and good reliability (Cronbach's *α* = 0.73–0.96) (18). QOL includes eight dimensions: vitality (VT), social functioning (SF), role-emotional (RF), mental health (MH), physical functioning (PF), role-physical (RP), bodily pain (BP), and general health (GH). The total scores are 100 points. The higher the scores, the better the QOL.

### 2.6 Statistical analysis

Data analysis was conducted via SPSS 22.0 software. The measurement data are represented by *x* ± *s*, with a *t*-test for intergroup comparison. Count data are represented by [*n* (%)], with *χ*^2^ test for intergroup comparison. To reflect the effect size of the intervention, Cohen's *d* was used, in which *d* < 0.2 is considered a very small effect size, 0.5 > *d* = > 0.2 a small effect size, 0.5 < *d* < 0.8 a medium effect size, and *d* < 0.8 a large effect size. The difference depicted statistical significance with *p* < 0.05.

## 3 Results

### 3.1 Comparison of general data between both groups

No statistical significance was depicted between both groups in terms of surgical time, intraoperative bleeding volume, gender, age, and BMI (*p* > 0.05; Table 1), indicating comparability.

**Table 1:** General data in both groups.

Groups	*N*	BMI (kg/m^2^)	Surgical time (min)	Intraoperative bleeding volume (ml)	Gender (male/female)	Age (years)
Control group	43	22.10 ± 3.17	65.98 ± 4.37	9.33 ± 1.24	23/20	38.85 ± 3.49
Responsibility group	43	22.74 ± 3.15	65.92 ± 4.56	9.47 ± 1.33	22/21	38.76 ± 3.33
*χ*^2^/*t*		1.388	0.538	1.394	0.047	0.435
*p*-value		0.172	0.594	0.171	0.829	0.666
95% confidence interval		−0.715 to −1.995	−1.975 to −1.855	−1.309 to −1.589	/	−1.553 to −1.373
Cohen's *d*		−0.202	0.013	−0.108	/	0.026

BMI, body mass index.

### 3.2 Comparison of postoperative recovery between both groups

The first postoperative mealtime, the first postoperative exhaust time, postoperative hospitalization time, and the first postoperative out-of-bed activity time in the responsibility group depicted depletion relative to those in the control group, indicating statistical significance (*p* < 0.05; Table 2).

**Table 2:** Postoperative recovery in both groups.

Groups	*N*	First postoperative mealtime (d)	First postoperative exhaust time (day)	Postoperative hospitalization time (day)	First postoperative out-of-bed activity time (h)
Control group	43	2.22 ± 0.14	2.19 ± 0.18	5.37 ± 0.44	5.30 ± 0.90
Responsibility group	43	1.72 ± 0.34	1.67 ± 0.12	4.19 ± 0.33	4.52 ± 0.83
*t*		9.522	11.536	12.278	3.175
*p*-value		<0.001	<0.001	<0.001	0.003
95% confidence interval		–0.612 to –0.387	–0.585 to –0.454	–1.347 to –1.013	–1.151 to –0.408
Cohen's *d*		1.923	3.399	3.034	0.900

### 3.3 Comparison of total satisfaction rate post-nursing care between both groups

The total nursing satisfaction rate in the responsibility group (42/43; 97.67%) was higher than that in the control group (36/43; 83.72%) post-nursing care, indicating a statistical difference (*p* < 0.05), as shown in Table 3.

**Table 3:** Total satisfaction rate post-nursing care in both groups.

Groups	*N*	Significant satisfaction	Satisfaction	Dissatisfaction	Total satisfaction rate (%)
Control group	43	16	18	9	34 (79.07%)
Responsibility group	43	35	7	1	42 (97.67%)
*χ* ^2^		/	/	/	7.242
*p*-value		/	/	/	0.007

### 3.4 Comparison of postoperative complications between both groups

The incidence of complications such as intestinal obstruction, incision infection, and abdominal abscess in the responsibility group 14 days post-surgery (4.65%) depicted depletion relative to that in the control group (18.6%), indicating statistical significance (*p* < 0.05; Table 4).

**Table 4:** Postoperative complications in both groups.

Groups	*N*	Intestinal obstruction	Incision infection	Abdominal abscess	Total complication rate (%)
Control group	43	4	3	1	8 (18.60%)
Responsibility group	43	1	1	0	2 (4.65%)
*χ* ^2^		/	/	/	4.074
*p*-value		/	/	/	0.044

### 3.5 Comparison of QOL scores between both groups

No statistical significance in QOL scores such as VT, SF, RF, MH, RF, RP, BP, and GH between both groups before nursing (*p* > 0.05). The QOL scores in both groups post-nursing care were higher relative to before, and those in the responsibility group were higher relative to controls, indicating statistical significance (*p* < 0.05; Table 5).

**Table 5:** QOL scores in both groups.

QOL	Nursing stage	Control group	Responsibility group	*t*	*p*-value	95% confidence interval	Cohen's *d*
VT	Before nursing	55.67 ± 4.36	55.48 ± 4.32	1.167	0.25	−0.190 to −0.936	0.043
	Post-nursing care	65.45 ± 6.54*	76.28 ± 6.25^*^^,^^#^	6.327	<0.001	8.087–13.570	−1.693
SF	Before nursing	63.44 ± 5.27	63.46 ± 5.18	1.105	0.275	−2.221 to −2.261	−0.003
	Post-nursing care	72.35 ± 6.47*	79.46 ± 7.24*^,#^	3.578	<0.001	4.165–10.060	−1.035
RE	Before nursing	65.12 ± 5.39	64.57 ± 5.25	0.416	0.679	−2.832 to −1.732	0.103
	Post-nursing care	76.42 ± 6.55	85.37 ± 7.55	4.076	<0.001	5.918–11.980	−1.266
MH	Before nursing	66.57 ± 5.47	66.54 ± 5.35	0.56	0.579	−2.350 to −2.290	0.005
	Post-nursing care	72.38 ± 6.35	86.42 ± 7.22	10.131	<0.001	11.120–16.960	−2.065
RF	Before nursing	60.83 ± 5.38	61.28 ± 5.46	1.689	0.099	−1.875 to −2.775	−0.083
	Post-nursing care	75.34 ± 5.26	80.45 ± 6.34	2.327	0.025	2.611–7.609	−0.877
RP	Before nursing	55.31 ± 4.39	55.28 ± 4.57	0.122	0.904	−1.952 to −1.892	0.006
	Post-nursing care	62.34 ± 5.53	72.83 ± 5.63	9.382	<0.001	8.097–12.880	−1.879
BP	Before nursing	63.67 ± 5.62	63.75 ± 5.47	0.636	0.528	−2.298 to −2.458	−0.014
	Post-nursing care	79.15 ± 6.22	85.56 ± 6.24	6.394	<0.001	3.738–9.082	−1.028
GH	Before nursing	76.98 ± 1.48	76.83 ± 1.49	1.092	0.281	−0.786 to −0.486	0.101
	Post-nursing care	84.02 ± 1.49	90.87 ± 1.34	24.35	<0.001	6.242–7.458	−4.834

QOL, quality of life; VT, vitality; SF, social functioning; RF, role-emotional; MH, mental health; PF, physical functioning; RP, role-physical; BP, bodily pain; GH, general health. *Note*: “*” *p* < 0.05 vs. before nursing (within-group comparison); “#” *p* < 0.05 vs. control group after nursing (between-group comparison).

## 4 Discussion

Appendicitis is one of the common diseases in surgery. Laparoscopic surgery for appendicitis possesses a high success rate and can effectively facilitate patients' recovery (19, 20). Nevertheless, any surgery exerts a certain stress influence, and postoperative nursing management needs to be strengthened. It is suggested that clinical medical technology is currently advanced, and postoperative clinical efficacy is excellent, whereas professional nursing after appendectomy is also very crucial (21). Thus, providing corresponding nursing interventions post-surgery exerts a vital role in clinical efficacy and clinical nursing satisfaction.

The vertical and horizontal hierarchical responsibility management nursing model strengthens the training of responsible nursing staff and refines nursing work contents based on the traditional nursing intervention model. Based on patients' condition, corresponding nursing measures are formulated to further elevate the quality of nursing, enhance the sense of responsibility of the nursing staff, and further improve the effectiveness of nursing management. The application of the vertical and horizontal hierarchical responsibility management nursing model in clinical practice can also actively adjust nursing strategies to meet patients' needs and facilitate early postoperative nutritional balance, and nutritional intervention can attenuate fat oxidation and overall protein metabolism, improve postoperative malnutrition, and facilitate patient recovery (22). Herein, the total nursing satisfaction rate in the responsibility group depicted elevation relative to that in the control group, and first postoperative mealtime, first postoperative exhaust time, postoperative hospitalization time, and first postoperative out-of-bed activity time in responsibility group depicted depletion relative to those in the control group, suggesting that application of vertical and horizontal hierarchical responsibility management nursing model in appendectomy patients can effectively elevate patients' satisfaction and facilitate patients' recovery. From a mechanistic perspective, the vertical and horizontal hierarchical responsibility management nursing model can elevate the nursing quality of nursing staff, better facilitate recovery of intestinal peristalsis function, and contribute to patient recovery early intestinal function (23).

The responsibility-based nursing model focuses on patients, and the application of responsibility-based nursing can provide comprehensive, systematic, and holistic nursing for patients' physical and mental health, effectively elevating the quality of nursing services. The application of the responsibility-based nursing management model to nursing work of various diseases in clinical departments has validated that such nursing intervention possesses high application quality in both disease intervention and department implementation (24). Herein, the incidence of complications such as intestinal obstruction, incision infection, and abdominal abscess in the responsibility group 14 days post-surgery depicted depletion relative to that in controls; QOL scores in both groups post-nursing care were higher relative to before, and those in the responsibility group were higher relative to controls. The above findings suggested that the application of the vertical and horizontal hierarchical responsibility management nursing model in appendectomy patients has elevated their QOL. This is because in the vertical and horizontal hierarchical responsibility management nursing model, nursing staff enhanced communication with patients to improve the accuracy of care and the patient's compliance, implemented continuous care to ensure the continuity and stability of the rehabilitation process, and strengthened psychological support, improving the psychological state and quality of life of patients. Consistently, Chen et al. (6) suggested that rapid rehabilitation surgical nursing interventions provided to patients after laparoscopic appendectomy can accelerate their postoperative recovery, reduce the occurrence of complications, and improve their sleep quality and nursing satisfaction. Moreover, Guo et al. (15) indicated that implementing a hierarchical management model positively impacts nursing quality within the nursing department and enhances patient satisfaction.

Our research has some limitations. Firstly, our sample size is relatively small, which may lead to deviations between the data results and the actual values. Secondly, our research was a single-center study, and the sample was not representative, which may not accurately reflect the characteristics of a broader population. Fourthly, our study only had a relatively short follow-up period. The effects of the vertical and horizontal hierarchical responsibility management nursing model on the long-term recovery of patients after appendectomy are currently unclear. Therefore, more multicenter, large-scale, and long-term studies should be conducted in the future to further verify our findings.

## 5 Conclusion

The application of the vertical and horizontal hierarchical responsibility management nursing model in appendectomy patients can improve clinical nursing efficacy and facilitate patients' recovery, which is worthy of clinical application.

## Data Availability

The datasets presented in this study can be found in online repositories. The names of the repository/repositories and accession number(s) can be found in the article/Supplementary Material.
